# Staple line reinforcement with nebulized cyanoacrylate glue in laparoscopic sleeve gastrectomy: A propensity score-matched study

**DOI:** 10.1515/med-2022-0426

**Published:** 2022-01-19

**Authors:** Gennaro Martines, Giovanni Tomasicchio, Arcangelo Picciariello, Rigers Dibra, Giuseppe Trigiante, Giuliano Lantone, Donato Francesco Altomare

**Affiliations:** Department of Emergency and Organ Transplantation (DETO), University of Bari “Aldo Moro”, Piazza Giulio Cesare, 11, Bari, Puglia, Italy; Department of Emergency and Organ Transplantation (DETO), University of Bari “Aldo Moro”, Piazza Giulio Cesare, 11, Bari, Puglia, Italy

**Keywords:** laparoscopic sleeve gastrectomy, leak, suture reinforcement, sealant

## Abstract

**Background:**

A dreaded complication of laparoscopic sleeve gastrectomy (LSG) is suture leak. The study aimed to assess the efficacy of the nebulized comonomer Glubran 2^®^ (*N*-butyl-cyanoacrylate + metacrylosysolfolane) applied to the LSG staple line.

**Methods:**

A propensity-matched comparison analysis was conducted in 125 patients undergoing LSG between 2017 and 2019. Groups included those treated with Glubran^®^ (group 1, *n* = 70) and those without Glubran^®^ treatment (group 2, *n* = 55).

**Results:**

There were differences in the mean body mass index (44.4 vs 43 kg/m^2^; *P* < 0.05) between the groups. There was a non-significant increase in the operative time for group 1 compared with group 2 (97 ± 8 vs 93.8 ± 10.7 min; *P* = 0.07), with a greater amount of estimated blood loss (94.5 mL vs 87.8; *P* < 0.01). There were more severe complications in group 2 over group 1 cases (8 vs 0%; *P* < 0.05), although postoperative bleeding did not differ between the two groups (1.4 vs 5.4%). There were no postoperative leaks in group 1 patients, but there were two leaks in group 2 cases with an increased length of hospital stay in patients with a leak.

**Conclusion:**

Glubran^®^ LSG support may reduce leak risk without increasing operating time.

## 1 Introduction

Since 1975, worldwide obesity has tripled in incidence affecting all age groups in a manner that has been described as a global pandemic [1]. Over a decade ago, global overweight and obesity have been estimated to be responsible for 3.4 million deaths, 4% of years of life lost, and nearly 4% of disability-adjusted life years [2], with the expected rise in obesity in the United States projected to cause a decline in the future population life expectancy [3]. Although the management of obesity requires a multidisciplinary approach that includes dietary and lifestyle interventions, there are consensus indications for bariatric surgery, which when indicated have proven very effective in weight loss and weight maintenance [4,5,6]. Currently, laparoscopic sleeve gastrectomy (LSG) is one of the most common operations for morbid obesity [7], proving relatively easy and quick to perform and markedly less complex than other procedures such as Roux-en-Y gastric bypass or biliopancreatic diversion [8,9]. Compared with Roux-en-Y gastric bypass, LSG requires less operating time and is accompanied by an easier learning curve and has 50% fewer complications [10].

With LSG, staple line problems remain a concern with reported leak rates ranging from 1.1 to 4.7% [11] and hemorrhage in up to 4% of the cases [12]. Both of these serious complications are technique dependent and are related to the degree of staple compression and the appropriateness of staple height selection. In each case, such a major perioperative complication has significant implications for mortality, morbidity, and health care costs [13]. There has been a considerable focus over the last decade on the possible risk factors for complications following an LSG, with particular emphasis on mechanisms for reducing suture-line leaks. These approaches have included varying the bougie size, altering the distance from the pylorus, and a variety of different staple-line reinforcement techniques [14]. Recently, the nebulized comonomer cyanoacrylate (*N*-butyl-cyanoacrylate [NBCA]  +  metacrylosysolfolane [MS], Glubran 2^®^, (GEM s.r.l. Viareggio [LU] – Italy) has been proposed as a novel gastric suture reinforcement material with Pilone et al. [15] demonstrating its efficacy when Glubran^®^ spray is combined with an omentopexy. This modified glue, which is widely used in surgery and endoscopy, in particular, in the emergency management of patients with upper gastrointestinal bleeding, results in an adhesive, hemostatic seal when nebulized and sprayed on tissues, which also acts as an antiseptic barrier against the most commons pathogenic agents [16]. We present a retrospective observational single-center study evaluating the efficacy of a nebulized cyanoacrylate seal in LSG in a cohort of morbidly obese patients, comparing these with the standard procedure in a control group.

## 2 Materials and methods

A dedicated electronic database was prospectively maintained of obese patients undergoing LSE in a tertiary referral center for bariatric management. The analysis examines patients managed in the unit between January 2017 and January 2019. Inclusion criteria were those patients over 18 years of age, with a body mass index (BMI) >40 or >35 kg/m^2^ with at least one comorbid condition, such as hypertension, dyslipidemia, or diabetes, who were considered medically fit for surgical intervention. Exclusion criteria were those patients who had any intraoperative evidence of a minor leak on testing, cases with other active gastric disease or patients with either an uncontrolled medical complaint, or an attendant psychiatric condition. These inclusion and exclusion criteria were developed in accordance with published international guidelines [17].

Patients were separated into two groups: group 1 managed with Glubran^®^ reinforcement and group 2, a control arm, where there was no staple-line reinforcement used. All procedures were performed by the same surgeon. Demographic data were collated including patient age, gender, weight (kg), BMI (kg/m^2^), and comorbidity. Intraoperative parameters collected included the operative time, the estimated intraoperative blood loss, and the open conversion rate. Postoperative parameters registered included the length of hospital stay (LOHS), the timing of removal of abdominal drains, and the incidence of hospital readmission within 30 days. Postoperative complications were recorded in accordance with the published Clavien–Dindo classification [18], including staple-line leakage and/or gastric fistula, postoperative hemorrhage, intra-abdominal sepsis/collection(s), and cardiopulmonary problems.

Permission for the conduct of this retrospective analysis was provided by the local hospital Ethics Committee. Informed consent was obtained from all individual participants included in the study. All investigations complied with the principles of the Declaration of Helsinki.

## 3 Surgical procedure

All patients provided informed consent for surgery after thorough explanation and counseling of the benefit:risk ratio. Under general anesthesia, patients were placed in a modified lithotomy position with a 10° reverse Trendelenburg tilt. Thromboprophylaxis was provided with elastic stockings during surgery along with the administration of LMW heparin, which was continued for 21 days postoperatively. A 5-mm trocar method was used to establish pneumoperitoneum at 15 mmHg. The omentum was dissected away from the great curvature of the stomach with an Harmonic Scalpel^®^ (Ethicon Endo-surgery, LLC, Guayanabo, PR, USA), beginning opposite the Crow’s foot (approximately 6 cm proximal to the pylorus) and reaching as far as the angle of His. Transection of the stomach was performed using a 60 mm linear Echelon^®^ stapler (Ethicon Endo-Surgery Cincinnati, OH) with sequential firing in the antrum of a green (4.1 mm staple height open, 2 mm closed) stapler cartridge, a yellow (3.25 and 1.5 mm) cartridge for the greater curvature, and a blue (3.5 and 1 mm) cartridge for the upper stomach.

To reduce the chances of intraoperative bleeding, we allowed a period of 30 s to elapse between the time the stapler was closed and when it was fired. Any staple-line bleeding was managed with diathermy coagulation. A 38 Fr. gastric bougie was passed and positioned against the lesser curvature to calibrate the final volume of stomach after transection. A standard intraoperative leak test was performed using methylene blue instilled via the bougie. Nasogastric tubes were not used. A thin layer (1 mL volume) of nebulized Glubran 2^®^ was applied with a laparoscopic nebulizer (GEM s.r.l. Via dei Campi, 2 – Viareggio, Italy) as a reinforcement of the staple line in group 1 cases. A 19 Fr. silicon drain (BLAKE ^®^ Ethicon USA) was placed alongside the staple line checking for the possibility of gastric rotation or any sign of tension in the stomach remnant. A Gastrografin^®^ (Bayer S.p.A., Leverkusen, Germany) study was routinely performed on the fifth postoperative day after which a fluid oral diet was commenced if there was no evidence of contrast leakage.

## 4 Statistical methods

Statistical analysis was performed with the SAS 9.1.3 (SAS Institute Cary NC) software. Continuous parameters were reported as frequencies, means ± standard deviation (SD), where appropriate. Categorical variables were recorded as numbers and percentages. The statistical analysis to compare the two groups was performed using the Student *t*-test where indicated with the homogeneity of variance between study groups verified with the *F* test. The Satterthwaite approximation was used to determine effective degrees of freedom in instances of unequal variance. Comparisons of categorical variables were performed by the Chi-square and Fisher’s exact test where appropriate. Group 1 and group 2 cases were not paired. As there were unequal sample sizes and a nonhomogeneous distribution of gender, weight, and BMI, propensity score matching was used to reduce the biases inherent in the analysis [19]. Propensity scores were calculated using a logistic regression model where age, gender, weight, BMI, and the number of comorbidities for each subject were the covariates and the treatment (Glubran^®^ or no Glubran^®^) was the dependent variable. The Greedy data matching algorithm was used to produce matched samples with balanced covariates. Within propensity scores, the Mahalanobis distance was used for correlation as a measure of the distance between points in the distribution. All *P*-values <0.05 were considered significant.

## 5 Results

There were 125 patients enrolled in the study (mean age: 42.6 years, min ÷ max: 19 ÷ 64). Of these patients, 70 were in group 1 with Glubran^®^ reinforcement (mean age: 43.5 years, min ÷ max: 20 ÷ 64) and 55 had no reinforcement and were in group 2 as controls (mean age: 41.4 years, min ÷ max: 19 ÷ 60). In group 1, there were 58 (83%) women and 12 (17%) men, whereas in group 2, there were more women (52 women [95%] and 3 men [5%], *P* < 0.05). Differences were noted between group 1 and group 2 in mean weight (117.1 kg, min ÷ max: 90 ÷ 160 vs 110.3 kg, min ÷ max: 94 ÷ 130, respectively; *P* < 0.01) and in mean BMI (44.4 kg/m^2^, min ÷ max: 34 ÷ 60 vs 43 kg/m^2^, min ÷ max: 37 ÷ 50; *P* < 0.05). The demographic features of the groups are shown in Table 1. The comorbidities of the patient cohort are shown in Table 2 with a difference between group 1 and group 2 in the incidence of diabetes (36 vs 60%, respectively; *P* = 0.01) and in respiratory ailments (69 vs 35% respectively, *P* = 0.003). There were no differences evident between the groups in patients with no comorbidities or with at least one comorbidity. All of the procedures were performed laparoscopically without the need for open conversion in any case.

**Table 1:** Demographic features of group 1 and group 2 patients (*n* = 125)

Parameter	Statistic	Glubran (*N* = 70)	Control (*N* = 55)	All subjects (*N* = 125)
Age (years)	Mean	43.5	41.4	42.6
	Min ÷ max	20 ÷ 64	19 ÷ 60	19 ÷ 64
	SD	10.6	10	10.3
	^*P*		0.2755	N.S.
	§*P*		0.6669	N.S.
Gender				
Male	*N* (%)	12 (17%)	3 (5%)	15 (12%)
Female	*N* (%)	58 (83%)	52 (95%)	110 (88%)
	*P*		0.0459	*P* < 0.05
Weight (kg)	Mean	117.1	110.3	114.1
	Min ÷ max	90 ÷ 160	94 ÷ 130	90 ÷ 160
	SD	15.7	8.5	13.4
	^*P*		0.0024	*P* < 0.01
	§*P*		<0.0001	*P* < 0.01
BMI (kg/m^2^)	Mean	44.4	43	43.8
	Min ÷ max	34 ÷ 60	37 ÷ 50	34 ÷ 60
	SD	4.7	2.8	4.1
	^*P*		0.0443	*P* < 0.05
	§*P*		0.0002	*P* < 0.01

Notes: ^The *P*-value is based on the *t*-test with unequal variances according to the Satterthwaite variation. § of *F* test significant where *P* <0.05.

All other *P*-values are based upon *t*-testing with equal variances.

**Table 2:** Comorbidities of the patient cohort

Parameter	Category	Statistic	Glubran (*N* = 70)	Control (*N* = 55)	All subjects (*N* = 125)
Comorbidity	None	*N* (%)	11 (15.7)	8 (14.5)	19 (15.2)
	At least one comorbidity	*N* (%)	59 (84.3)	47 (85.5)	106 (84.8)
		*P*-value*		0.8566	N.S.
	Cardiovascular	*N* (%)	42 (60.0)	30 (54.5)	72 (57.6)
	Diabetes	*N* (%)	25 (35.7)	33 (60.0)	58 (46.4)
	Arthropathy	*N* (%)	13 (18.6)	7 (12.7)	20 (16.0)
	Respiratory	*N* (%)	48 (68.6)	19 (34.5)	67 (53.6)

*Chi-square test.

The intraoperative data are summarized in Table 3. The mean operative time was greater in group 1 cases when compared with group 2 cases (97 ± 8 vs 93.8 ± 10.7 min, respectively; *P* = 0.07), although this did not reach significance. There was a significantly greater amount of estimated blood loss in group 1 patients when compared with group 2 cases (94.5 ± 30 vs 87.8 ± 29.8 mL, respectively; *P* < 0.01). No intraoperative complications were recorded, and there was no perioperative mortality. Table 4 shows the list of postoperative complications according to Clavien–Dindo grade. The percentage of patients with more severe complications was significantly higher in the group 2 cases when compared with group 1 patients (9 vs 0%, respectively; *P* < 0.05). Table 5 shows the specific complications recorded in the groups. There were no differences recorded between the two groups concerning the frequencies of postoperative bleeding, intra-abdominal abscess, or cardiopulmonary complications. There were three (5.4%) of the patients from group 1 and one (1.4%) of the patients from group 2 who had postoperative bleeding with each successfully managed by conservative means and with no requirement, in any case, for transfusion. The intra-abdominal abscesses in the group 2 caused by acute infection of blood were successfully treated with surgical intervention under local anesthesia. There were no postoperative leaks in the Glubran^®^-treated group 1 patients, whereas there were two leaks in the group 2 control cases. Three of these two leaks were evident between the seventh and the eighth postoperative days. All of the patients with a leak were successfully managed with laparoscopic peritoneal lavage and insertion of a silicon drain to the subdiaphragmatic space. Abdominal drains were removed on average at 7 days in each group (*P* = 0.32) with no significant differences noted between the groups in the median LOHS (group 1, 6.2 ± 0.7 days vs group 2, 8 ± 7 days; *P* = 0.07).

**Table 3:** Intraoperative parameters

Parameter		Glubran (*N* = 70)	Control (*N* = 55)	All subjects (*N* = 125)
Operative time (min)	Mean	97.0	93.8	95.6
	Min ÷ max	82 ÷ 117	80 ÷ 120	80 ÷ 120
	S.D.	8.0	10.7	9.4
	^*P*		0.0711	N.S.
	§*P*		0.0203	*P* < 0.05
Estimated intraoperative	Mean	94.5	79.3	87.8
Blood loss (mL)	Min ÷ max	50 ÷ 150	50 ÷ 150	50 ÷ 150
	SD	30	27.4	29.8
	^*P*		0.0041	*P* < 0.01
	§*P*		0.4941	N.S.

Notes: ^*P*-value is based on *t*-test with unequal variances (Satterthwaite approximation) §*P* < 0.05).

All other *P*-values are based on *t*-testing with equal variances.

**Table 4:** Postoperative complications according to grade

	Group 1 (%)	Group 2 (%)	Total (%)	*P*-value
No complication	63 (90)	48 (82.2)	111 (88.8)	0.037*
Grade I	5 (7.1)	0 (0)	5 (4)	0.066
Grade II	2 (2.9)	2 (3.6)	4 (3.2)	1
Grade III				0.034*
a	0 (0)	3 (5.4)	3 (2.4)	
b	0 (0)	2 (3.6)	2 (1.6)	

*Chi-square test. Complications were listed according to the Clavien–Dindo classification [18].

**Table 5:** Specific postoperative complications

	Glubran (*N* = 70)	Control (*N* = 55)	Total	*P*-value (*N* = 125)
Postoperative hemorrhage (%)	1 (1.4)	3 (5.4)	4 (3.2)	0.319
Leak (%)	0 (0.0)	2 (3.6)	2 (1.6)	0.191
Intra-abdominal abscess (%)	0 (0.0)	3 (5.4)	3 (2.4)	0.082
Cardiopulmonary complication	2 (2.8)	3 (5.4)	5 (4)	0.653

*P* values are determined with the Chi-square test.

The Greedy data matching algorithm identified 49 pairs with the best matching for age, gender, weight, BMI, and comorbidities. No significant differences were found for any intraoperative or postoperative variable. There were no differences between the groups in the time to drain removal or in hospitalized days, but in patients with a leak, the hospitalization was 10 days longer than in those without a leak (median of 6 days). The incidence of severe complications (Clavien–Dindo grades III–IV) was significantly higher in group 2 patients.

## 6 Discussion

We performed a propensity-matched analysis in patients undergoing a LSG for morbid obesity, comparing patients with or without Glubran^®^ (GEM, s.r.l., Italy) reinforcement of the gastric staple line. There were significantly fewer Clavien–Dindo grade III/IV postoperative complications and fewer suture leaks in the treated group with a substantial increase in the LOHS if a leak occurred. The LSG procedure was initially introduced as a first-step in super obese patients (BMI > 50) and as an alternative to the more complex surgeries such as gastric bypass or biliopancreatic diversion [5]. Over recent years, LSG has gained worldwide popularity with a declaration in 2012 by the American Society for Metabolic and Bariatric Surgery (ASMBS) that it was the acceptable primary bariatric surgical option [20]. Since then, LSG is the most commonly performed low-risk surgical procedure, which consistently provides durable weight loss and significant improvements in medical comorbidities [21]. The position of the ASMBS has recently been updated confirming the value of LSG in obesity treatment [22] where, in comparison with Roux-en-Y gastric bypass, the initial weight loss appears similar as does the improvement in weight-related comorbid conditions and hospital readmission rates, but there were far fewer major postoperative complications [23].

A leak from a staple line remains the most important and dangerous complication associated with LSG, resulting in prolonged hospitalization, an impaired clinical outcome, and increased health care costs [13,20,24]. Assessment of the available literature shows a leak rate varying between 1.5 and 4.2% with a reported mortality of 9% among leak patients overall [25]. Our reinforced leak rate is in keeping with previously published studies [26], with the leaks generally detectable by the fourth postoperative day. Intraoperative methylene blue testing, however, was not uniformly predictive where Sethi et al. [27] have shown a poor sensitivity but a high specificity of the test. Given the low incidence of a leak, the overall clinical utility of such intraoperative testing is questionable [28] particularly when there has been a concern that the stress imposed on the staple line during testing may actually contribute to weakness [29]. Given that the majority of leaks occur several days after surgery, most probably, as a result of tissue ischemia or gastric wall hematomas, intraoperative testing might only be of benefit in a staple misfire. Over time, there have been improvements in surgical technique, which have reduced leaks with less risk of thermal injury, better staple height selection, choice of an adequate larger bougie size leading to less risk of narrowing, routine takedown of the short gastric vessels, and complete mobilization of the fundus [30].

Numerous studies have defined the risk factors associated with leaks, identifying as significant predictive markers: older age, male gender, preoperative sleep apnea, the bougie size, the distance from the pylorus, the level of surgical expertise and the type of staple line reinforcement used [11,31,32]. Specifically concerning the issue of staple-line buttressing, an expert international panel surveying surgeons who had personally performed >1,000 cases compared bariatric practice at two time points, 2011 and 2014 [21]. In both surveys, expert surgeons reported that buttressing of staple lines was acceptable with a higher percentage of acceptance in a larger number of surgeons questioned in the later survey (77.3% of 23 surgeons questioned in 2011 vs 81.4% of 120 surgeons questioned in 2014; *P* < 0.001). There is a consensus regarding the use of the appropriate staple height for the different elements of the sleeve gastrectomy where a greater height is used for the antrum and the thicker part of the stomach. There is, however, still debate around the optimal technique for staple line reinforcement and the need to oversew, with different opinions between identified experts and bariatric general surgeons [20,33,34,35].

In a systematic review by Gagner and Kemmeter [13] comparing five different staple-line reinforcement techniques that included simple oversewing, tissue sealants, bovine pericardial strips, and no reinforcement, there was a significant reduction in leaks when an absorbable polymer membrane was used. By contrast, in a prospective randomized study reported by Carandina et al. [34], no benefit was evident from supplementing either an imbricated or a nonimbricated running suture with fibrin glue. In animal studies, buttressing results in a higher bursting strength [36] with the material that is used as a bolster capable of more evenly distributing the staple pressure over a wider surface area [37]. Providing that the correct staple heights have been selected for thicker tissues, buttressing will improve the staple compression [38]. The expectation of a staple-line benefit depends upon the timing of a leak following an LSG, where typically an ischemic event occurs at 5–7 postoperative days, whereas leaks at 48 h are mechanical in nature. There would be an apparent rationale for buttressing or other forms of staple-line reinforcement that are applicable to these earlier types of leak. In this respect, the addition of Glubran^®^ and its derivative Glubran 2^®^ (NBCA + MS), which both take advantage of rapid polymerization upon contact with water and blood, would appear to support this approach.

*In vivo* applications of cyanoacrylate evidenced an excellent hemostatic and adhesive properties in bonding biologic tissues and realized resistant glutting of tissues without alteration of the elasticity and preserving its integrity even in high tensile levels [39]. Furthermore, the hemostatic effect could contribute to prevent the hematoma formation on the staple line, and the nebulized form of the sealant allows an even distribution, preventing delayed bleeding and microperforation throughout the staple line. All these properties could play a role in the reinforcement of the staple line, reducing the risk of leak.

The use of a cyanoacrylate for specific use in LSG is a logical translation from other surgical procedures [40] as well as from successful use in the endoscopy room [41] and the radiology suite [42].

Our study has several limitations. First, the retrospective design potentially leads to analytic bias, second, there were only small numbers with unequal sample sizes, and third, there was no comparison with other staple line reinforcement methods. A propensity-matched score analysis, which obviated some of the analytical challenges, suggests an advantage for nebulized Glubran^®^ reinforcement of the staple line in obese patients undergoing LSG in the prevention of suture leaks. The addition of Glubran^®^ did not increase operative time and was not associated with postoperative complications. A future prospective, multicenter randomized controlled study that compares Glubran^®^ with other staple-line reinforcement techniques is indicated.

## 7 Conclusion

Since the LSG was first introduced, there has been a range of techniques designed to reduce the dreaded complication of a staple line leak. The present study adds to the available literature suggesting that support with nebulized cyanoacrylate (Glubran^®^) is preventative of leaks and can reduce postoperative morbidity.
